# The role of chronic pain and pain anxiety in delay discounting of pain and monetary losses

**DOI:** 10.1038/s41598-023-46378-4

**Published:** 2023-11-06

**Authors:** Wojciech Białaszek, Szymon Mizak, Paweł Ostaszewski, Przemysław Bąbel

**Affiliations:** 1 Institute of Psychology, DecisionLab: Center for Behavioral Research in Decision Making, SWPS University, Chodakowska 19/31, 03-815 Warsaw, Poland; 2 Institute of Psychology, Pain Research Group, Jagiellonian University, Romana Ingardena 6, 30-060 Kraków, Poland

**Keywords:** Psychology, Human behaviour, Risk factors

## Abstract

Pain may alter intertemporal decisions by modifying the value of pain-related outcomes. For example, a person with chronic back pain may be faced with two choices: undergo surgery that could provide long-term relief but would involve additional short-term pain and discomfort during recovery; or continue living with the chronic pain and avoid the surgery, thus leading to overall deteriorated health. Such choices are well captured by delay discounting, which is defined as the decline in the subjective value of an outcome as the delay of its receipt increases. We investigated general pain anxiety and delay discounting of monetary losses and pain in 255 individuals with and without chronic pain. We found that people with chronic pain tend to discount the value of pain outcomes more than those without chronic pain, suggesting that chronic pain may contribute to impulsivity in decision-making related to pain. Moreover, the effect of chronic pain on delay discounting was mediated through general pain anxiety. This result, however, should be taken with caution, because the effect sizes were small, and the path model was underpowered. In conclusion, people with chronic pain might be more likely to prioritize avoiding immediate discomfort and may undervalue the potential long-term benefits of actions that could alleviate their pain in the future.

## Introduction

A better understanding of the behavioral and cognitive aspects of pain is essential to develop effective and evidence-based interventions^1^. Chronic pain (CP) remains a challenge for pain clinicians^2,3^, and identifying decision-making mechanisms in this state is crucial for effective pain management as chronic pain can impair decision-making, including intertemporal decisions and decisions related to treatment (e.g., analgesic drug usage).

Delay discounting (DD) refers to the tendency for delayed outcomes to have less subjective value than more immediate ones^4,5^. Steeper discounting, indicative of impulsivity, involves preferring smaller immediate gains over larger delayed ones and delaying punishment or losses. This behavior can lead impulsive individuals to avoid minor medical procedures with less immediate pain, despite the risk of future complications. It has been shown that steeper DD is significantly associated with increased risk of maladaptive behaviors, such as tobacco use or opioid addiction^6–8^. Compared to people who demonstrate self-control, impulsive people will avoid immediate pain (e.g., by abusing opioids) because they perceive future outcomes as less aversive.

Previous studies combining pain and DD have focused primarily on constructing a theoretical model of pain-related decision making^9–11^ and DD that can be used to explain pain-related behavior, such as the impact of pain anticipation on affect^12^ and problematic behaviors, e.g., abuse of opioids^13^. Wakaizumi et al.^14^ have shown a direct link between CP and changes in discounting rate on both the behavioral and neural levels. Although no direct differences in DD between pain-free people and those experiencing chronic pain were found (see also Mistretta^15^), it was indicated that the intensity of the experienced pain is associated with steeper discounting of monetary rewards and a change in brain activity in areas associated with DD, compared to the brain activity of healthy controls. These findings indicate that the severity of CP can cause behavioral changes that are crucial for therapy planning; however, they leave numerous questions about the context of the relationships being studied. Moreover, the findings are mixed. In contrast to the mentioned studies^14,15^, which in terms of discounting rates of monetary and pain outcomes show no direct differences between those who do or do not experience CP, a study by Herman and Stanton^16^ revealed that chronic pain leads to short-sighted, more-impulsive decisions in the domain of monetary gains but not monetary losses.

With only limited and mixed empirical findings, the current state of knowledge does not allow us to clearly predict the relationship between CP and discounting losses. However, from the theoretical perspective, negative visceral factors, including pain, are thought to drive myopic decisions and result in impulsive choices^17,18^. Therefore, we hypothesize that CP sample will discount monetary and pain outcomes more steeply than a pain-free group.

In a study by Tompkins et al.^13^ participants completed four delay discounting tasks that assessed choices between immediate smaller vs. larger delayed money or pain, framed as either gains or losses. It was found that participants experiencing chronic pain and with high opioid misuse propensity were discounting monetary losses and a possibility of additional pain in the future more steeply than individuals at lower risk of opioid misuse experiencing chronic pain. These results in the domain of losses, not gains, supported by the notion that discounting of monetary and nonmonetary outcomes is positively correlated^19^, prompted us to hypothesize that discounting of money and pain will also correlate in our study.

The fear-avoidance model of pain, assumes that people who experience a higher level of fear of pain tend to avoid pain more often and—as a result—experience more pain^20^. The role of variables such as anxiety level^14^ remains unclear in this context, although it is related to the intensity of pain^21,22^ and the steepness of DD^23^. In line with these findings and the model, we hypothesize that chronic pain patients who experience a higher level of anxiety will tend to discount pain more, i.e., they will avoid immediate pain at the expense of longer-lasting later pain. These findings, led us to hypothesize a mediating role of pain anxiety in the relation between experiencing CP and delay discounting.

In order to understand the relationship between CP and DD more fully, there is a need to address these concerns, emphasizing the role of pain anxiety and differences in DD between those who experience CP and those who do not. The aim of the current study is thus to examine (1) the relationship between experiencing CP and the discounting of monetary and pain-related losses; (2) the relationship between discounting of different outcomes; and (3) pain anxiety as a possible mediator by which the experience of CP affects discounting.

## Results

CP was reported by 68 participants (CP group), and 182 did not report CP (Control group; five participants did not give a response). Within the CP group, the most frequently reported types of pain were back pain (33.8%), head pain (20.6%), and joint pain (7.4%). The remaining CP participants reported pain in other body parts (22.1%) or pain in multiple parts (16.2%). On average, the CP group had experienced pain for M = 5.42 years (SD = 5.2); on a scale from 0 to 10, its mean level of pain intensity was M = 4.16 (SD = 2.35), and unpleasantness was M = 4.43 (SD = 2.49).

The first set of analyses examined whether CP was related to how steeply delayed monetary loss or delayed pain are discounted. Preliminary comparisons between the CP group and the Control group found no group differences (CP: M = 0.425, SD = 0.298; Control: M = 0.470, SD = 0.352) in the level of delay discounting of monetary loss [t(135.18) = 0.97, *p* = 0.334, *d* = 0.131]; however, the groups differed in the discounting of delayed pain [t(123.72) = 6.172, *p* < 0.001, *d* = 0.870]. When pain was discounted in delay, the CP group had smaller AuC (M = 0.359, SD = 0.348) than the Control group (M = 0.678, SD = 0.374). Therefore the CP group exhibited grater preference towards delayed pain in comparison to the Control group. It is important to note that there was a significant difference in age between the two groups [t(70.491) = 7.865, *p* < 0.001, *d* = 1.687], with the CP group being significantly older (M = 42.42; SD = 16.78) than the Control group (M = 26.02, SD = 5.05). Analyses showed no differences in gender composition between the two groups [χ^2^ (1, N = 236) = 1.496, *p* = 0.221]. As hypothesized, the correlation between delayed monetary loss discounting and delayed pain discounting was positive and statistically significant (*r* = 0.382, *p* < 0.001). In the CP group, there was a significant relationship between current pain intensity and the AuC measure for monetary DD (r = − 0.272, *p* = 0.028), but not for pain DD (r = 0.026, *p* = 0.837) or current pain unpleasantness (for monetary DD: r = − 0.121, *p* = 0.338; pain DD: r = 0.030, *p* = 0.811).

Because the CP group was significantly older than the Control group, we decided to use propensity matching to match the groups by age (a possible confounding variable) using the nearest-neighbor matching method^24,25^. Due to large differences in age, out of the 180 participants in the Control group and the 66 in the CP group, the sample of matched participants consisted of 43 participants in each group. After matching, there were no group differences in age [t(79.847) = 1.300; *p* = 0.197, *d* = 0.280; CP group: M = 33.070, SD = 9.282; Control group: M = 30.721, SD = 7.360] or gender composition (equal number of males and females in both groups).

After controlling for the confounding effect of age, the subsequent analyses revealed the same pattern as the earlier results, in which there was no significant difference in the discounting of delayed monetary loss between the CP group (M = 0.441, SD = 0.319) and the Control group (M = 0.476, SD = 0.383) [t(75.864) = 0.453, *p* = 0.652, *d* = 0.101], but the group difference remained statistically significant for the discounting of delayed pain [*t*(78.844) = 2.810, *p* = 0.006, *d* = 0.625], in which the CP group had a smaller mean area under the discount curve (M = 0.439, SD = 0.381) compared to the Control group (M = 0.679, SD = 0.388). These analyses showed that CP is associated with a steeper discounting of future pain and therefore higher impulsivity related to pain. Importantly, the relationship between monetary and pain AuCs was still significant (*r* = 0.360, *p* < 0.001). Within the CP group there was no significant relationship between current pain intensity and the AuC measure (for monetary DD: *r* = − 0.184, *p* = 0.249; pain DD: *r* = 0.022, *p* = 0.892) or between current pain unpleasantness and the AuC measure (for monetary DD: *r* = − 0.021, *p* = 0.897; pain DD: *r* = 0.133, *p* = 0.406). These relationships suggest that the observed differences in discounting may not be attributable to current pain intensity or its unpleasantness.

**Figure 1:**
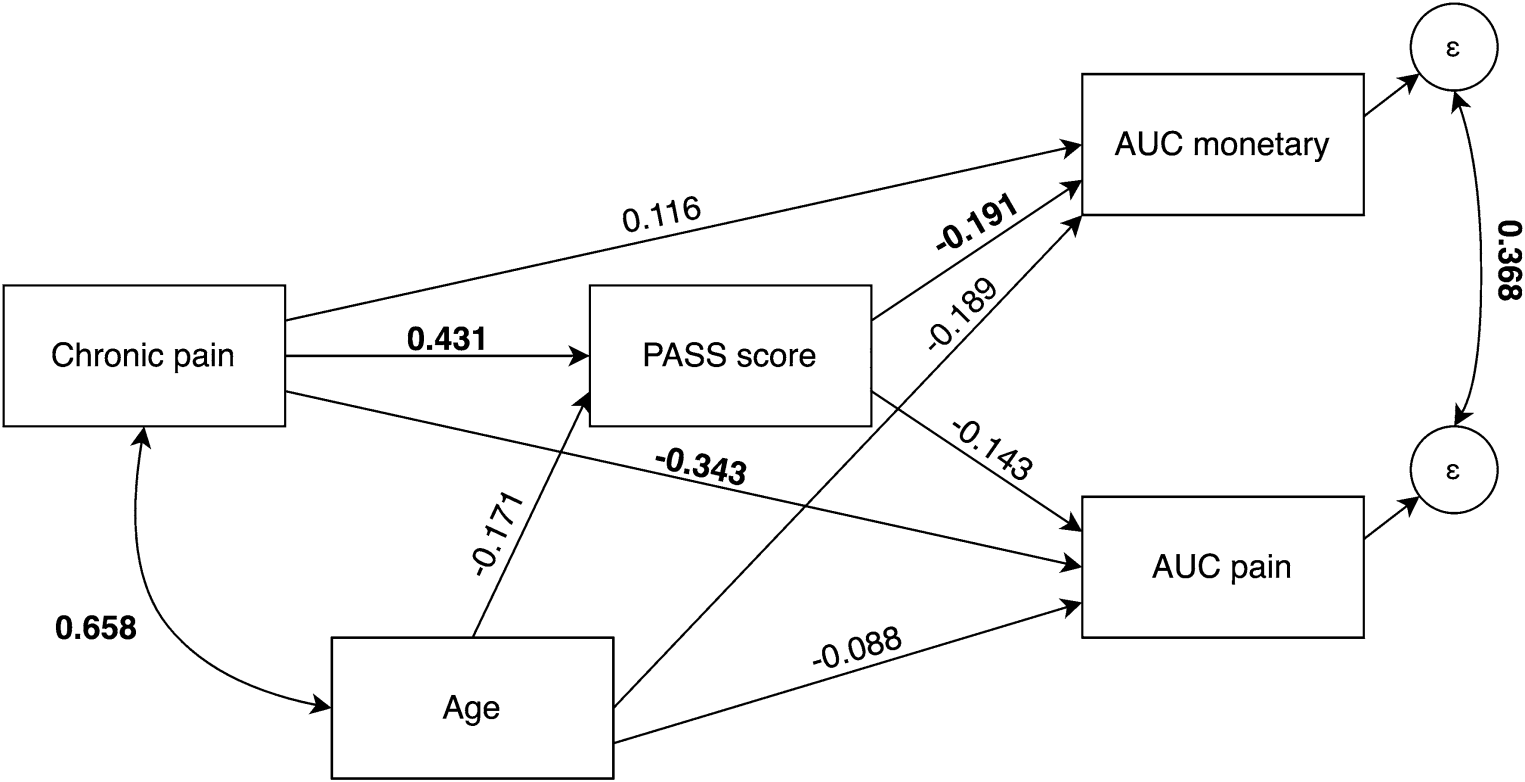
Model depicting the relationship between chronic pain, PASS general score, age and discounting measures. The paths are accompanied by their standardized coefficients (bold indicates coefficients that are significant at the *p* < 0.05 level).

A path model was then fitted to the full sample data to simultaneously describe the relationships between chronic pain, age, PASS score, and AuCs in one model. The proposed model and its standardized coefficients can be seen in Fig. 1. Due to the cross-sectional nature of the study, it should be noted that the proposed paths are merely a theoretical proposition; their directions cannot be tested nor falsified using the collected data. Thus, we do not interpret any coefficients in causal terms.

The estimated model coefficients again indicated that there was a relationship between CP and pain AuC (*γ* = − 0.343, 95% CI [− 0.562, − 0.124], *p* = 0.002), but not between CP and monetary AuC (*γ* = − 0.116, 95% CI [− 0.159, 0.391], *p* = 0.408). The coefficients’ estimates also indicated a strong positive relationship between CP and score on the PASS scale (*γ* = 0.431, 95% CI [0.214, 0.648], *p* < 0.001). In addition, the analysis showed a weak negative relationship between the PASS scale score and AuC for financial losses (*γ* = − 0.191, 95% CI [− 0.331, − 0.050], *p* = 0.008). We conducted hypothesis tests based on bootstrapped standard errors to test whether the indirect effects between CP and the measures of discounting by PASS score were significantly different from zero. In both cases, the standardized coefficients for the indirect effects were close to zero (pain AuC: *γ* = − 0.062, 95% CI [− 0.123, 0], *p* = 0.05; monetary AuC: *γ* = − 0.082, 95% CI [− 0.158, − 0.006], *p* = 0.033). Hypothesis tests showed that, in the case of both indirect effect associated with pain or monetary discounting, the effects could be considered significant; however, the effect sizes indicated marginal importance. In line with previous analyses, the covariance of the residuals for the two types of discounting (*γ* = 0.368, 95% CI [0.253, 0.483], *p* < 0.001), as well as covariance between CP and age (*γ* = 0.658, 95% CI [0.274, 0.743], *p* < 0.001), turned out to be positive, thus indicating that these pairs most likely share common variance, which could be further explained by variables that were not measured within this study.

## Discussion

CP can affect people’s behavior and cognition^14,26^. To our knowledge, this study is the first to investigate the link between experiencing CP or not and delay discounting of monetary losses and pain. These processes, despite being partially similar (as shown by the positive correlation between them), are in fact largely independent of each other. In particular, our study demonstrated a direct relationship between CP and the discounting of delayed pain, in which CP was associated with steeper discounting of pain, but not with a steeper discounting of monetary outcomes. From the perspective of our results, being in a state of chronic pain is related to shift in preferences away from choosing smaller (shorter pain duration) and sooner discomfort towards larger (longer pain duration) and later pain-related outcomes. CP patients are less willing to accept pain that is instant, thus possibly giving rise to avoidance.

Similarly to previous research^15,16^, we did not observe a direct relationship between CP and the discounting of monetary losses. However, in light of results that show that the discounting of losses and gains are, to a considerable extent, independent processes^27,28^ between which there no significant correlation^29^, this result does not necessarily contradict the results of Wakaizumi et al.^14^, and Herman and Stanton^16^, especially in the context of the observed mediation effects.

According to the current literature, the steeper discounting of pain can be explained by several mechanisms. For example, it can be hypothesized that chronic pain leads to a dysregulation of the emotional and attentional mechanisms responsible for pain processing, including those involved in decision-making^26^. A higher level of anxiety means a larger pool of cognitive and attentional resources is needed to achieve behavioral control. This limitation contributes to a more impulsive decision-making process in the context of pain-related outcomes. This mechanism may be one by which CP affects behaviorally manifested impulsivity, thus contributing to the occurrence of maladaptive behaviors.

Although the indirect (mediating) effects of the general pain anxiety score that mediates the relationship between the chronic pain and delay discounting of pain or monetary outcomes are significant, the effect sizes of these mediations can be interpreted as marginal. General pain anxiety constitutes only one possible mechanism by which the experience of chronic pain affects the subjective value of delayed outcomes. Further research should focus on theoretical models that would capture other theoretically driven mediators; alternatively, there should be a focus on common causes of the observed effects that were not identified or measured by the proposed model. In our study, the covariance of the residuals for the two discounted commodities is positive, pointing to the fact that the common variance could be further explained by variables that were not measured within this study. Analysis using a path model confirmed the effects observed in previous analyses and showed that the relationship between CP and pain discounting is also observed when anxiety is controlled for. This does not prove a direct relationship, of course, and future research should include potential common causes as well as potential mediators in the model. Yet, our analysis suggests that the relationship cannot be explained by pain-related anxiety levels alone and indicates either a direct or a more complex relationship. For example, Mistretta et al.^15^ found a significant moderating role of pain catastrophizing on the relation between delay discounting of monetary losses and chronic pain. Participants who were pain-free and were low on pain catastrophizing discounted monetary losses more in comparison to the CP group and those who were high on pain catastrophizing.

The role of pain anxiety revealed by our study is in line with the fear-avoidance model of pain. This model posits that individuals with elevated fear towards pain are more likely to evade pain, thereby paradoxically experiencing increased pain in the long run^20^. Similarly, our results show that chronic pain patients who experience a higher level of anxiety tend to discount pain more, i.e., they avoid immediate pain at the expense of longer-lasting later pain.

Our study is not without limitations. The first is related to the finding that the perception of pain (e.g., the pain threshold) changes with age^7,30^, as does delay discounting^31^. However, it is hypothesized that the latter stabilizes in young adulthood^32^. Further research should consider more homogeneous groups or use a global approach, including measures such as age, income, and health status as controlled variables in analyses. For example, Herman and Stanton^16^ concluded that people with chronic pain made more myopic decisions for monetary gains, not losses. This is not a straightforward result because initial analyses on discounting gains and losses pointed to differences in both domains. Only after considering socioeconomic and psychological covariates differences in discounting of gains remained statistically significant, but the result was not statistically significant in the domain of losses. Indirectly, this could mean that, although we did control only for sex and age, our sample was homogenous, without additional factors contributing to this relationship. Furthermore, when choosing homogeneous groups, it would be worthwhile to assess medication and therapeutic adherence because discounting processes provide explanatory mechanisms for maladaptive behaviors^33,34^. The second limitation concerns the proposed way in which our study captures seemingly causal relationships. In fact, from the perspective of the methodology used in this study, we could not assume that chronic pain is the cause of steeper discounting of pain. An alternative explanation for the observed effect might be that the degree of pain discounting affects CP. This may be viewed from the perspective of the relationship between the discounting rate and a range of health-related behaviors. More pronounced discounting is associated with an unhealthy diet^35^, smoking tobacco^36^, drug and alcohol use^37^, less frequent exercise^38^, and fewer health-oriented behaviors in general^38^. Therefore, more-impulsive individuals may be more likely to experience health problems^38^. This may lead to the conclusion that there may actually be a reverse cause-effect relationship between the amount of discounting and CP, where a higher level of behaviorally manifested impulsivity is a risk factor for CP due to negligence with respect to prevention or even treatment. The absence of a direct relationship between monetary discounting and CP indicates that the steeper discounting of delayed pain may be the effect of CP rather than the cause of it. In situations in which the degree of discounting would be thought to impact CP, we would also expect a direct relationship between CP and monetary discounting. The third limitation of our study is that some comparisons, in the tested path model (see Data analysis section) are underpowered. The sample in this study was a convenience sample, however, unlike in other studies^13,15,16^, where participants were recruited online, we directly recruited subjects.

The results of our study contribute to understanding the mechanism of pain-related impulsivity. Those suffering from CP show steeper delay discounting or are prone to opioid misuse^13,14^. In particular, as suggested by Tompkins et al.^13^, the discounting of pain and monetary losses (not gains) is associated with the misuse of drugs. It is also possible that excessive discounting can contribute to poor medication or therapeutic adherence^33,34^. The present study shows that people with CP are more impulsive than those without this condition, at least when outcomes are pain-related. Steeper delay discounting of pain in the CP group means an elevated probability of immediate pain avoidance, which may lead to maladaptive behaviors such as drug misuse. The possibility of more frequent engagement in risky and harmful behaviors could be addressed in pain-oriented therapeutic programs by monitoring risk and, if necessary, by introducing appropriate therapeutic interventions to prevent maladaptive behaviors, including opioid misuse.

## Methods

### Participants

This study included 255 participants (215 females, 39 males), who ranged in age from 20 to 75 years (M = 30.38, SD = 12.05; one person did not report gender and three did not report age). Participants in the control group were recruited from the community using snowball sampling and were examined in a university behavioral laboratory; the CP group consisted of patients recruited at healthcare facilities (specializing in the treatment of pain) and examined there. All participants in our study were adults, defined as individuals aged 18 years and older. The inclusion criteria for the CP group mandated that participants experience pain lasting longer than three months. Conversely, the Control group consisted of participants who declared not experiencing chronic pain. All participants were surveyed individually using a pen-and-paper form.

Participants were informed that they were taking part in a study on pain and decision making; they gave their written informed consent after reading the description of the study. They were also informed that they could withdraw their consent at any time without providing a reason. The study protocol was approved by the Research Ethics Committee at the Institute of Psychology (Jagiellonian University, Kraków, Poland) and is in accordance with the Helsinki Declaration of 1975, as revised in 1983.

### Measures

#### Basic demographics and pain-related measures

All participants were asked to provide their age and gender and whether or not they experience CP. Those who said they did experience CP were asked about the pain’s location and duration (in an open-ended question). They also rated its current intensity on an 11-point numeric rating scale (NRS) ranging from 0 = “no pain” to 10 = “the most intense pain imaginable”; they also rated its current level of unpleasantness on an NRS, with 0 = “not at all unpleasant pain” and 10 = “the most unpleasant pain imaginable.”

#### Delay discounting

**Figure 2:**
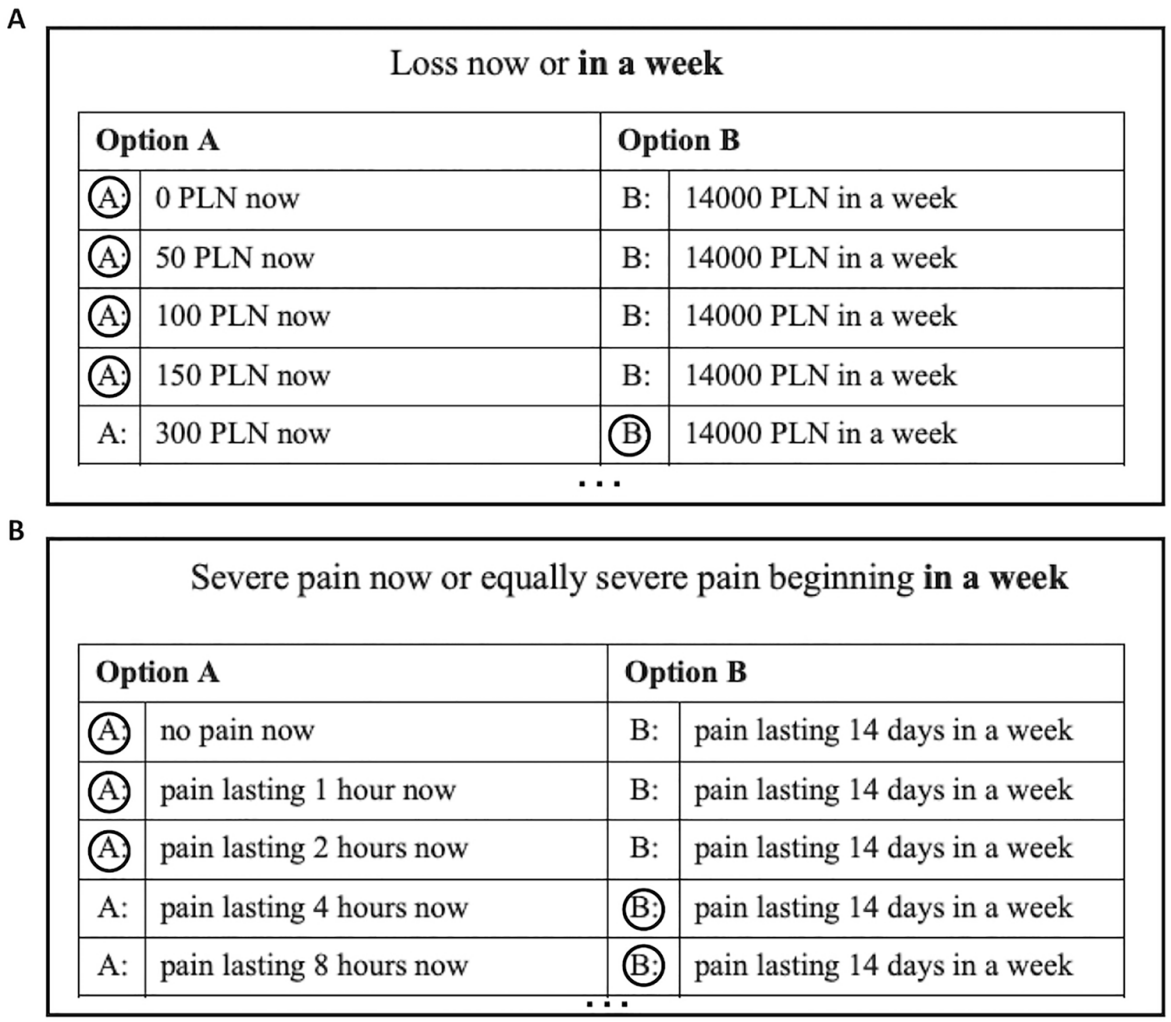
Sample excerpts of discounting inventories for (**A**) monetary and (**B**) pain conditions with sample answers. The full table consisted of 20 rows. The last selected choice in column A served as an approximation of the indifference point. Subsequent delay conditions were presented as consecutive tables on the following pages (5 for each domain condition).

The participants completed a monetary delay discounting task and a pain delay discounting task^9^. The two discounting tasks were administered using the pen-and-paper method and were presented in a counterbalanced order. The tasks used a fixed-sequence titration method in a mixed experimental design, with a loss outcome of 14,000 PLN (Polish zloty) in delay (1 PLN equaled approximately 0.26 USD at the time of the study) in the monetary discounting condition, and pain lasting for 14 days in the pain discounting condition. The subjective value of the monetary loss and the duration of pain was assessed after five delay durations (presented in ascending order): 1 week, 1 month, 1 year, 5 years, and 15 years. Participants were asked to choose hypothetical monetary losses for each delay from pre-set lists of losses. The alternatives were shown in two columns that presented options A and B on a single page, and the delay to the outcome (monetary loss or pain) was specified at the top of each page. For the monetary task, the left-hand column containing option A included rows with an immediate loss of 0 PLN to 14,000 PLN (in 20 increments), while the right-hand column included rows with a fixed loss of 14,000 PLN and a specified delay. The participants indicated their preferences by circling their chosen option in each row until their preferences shifted from option A to B. For example, the first choice in the shortest delay was (A) losing 0 PLN now, or (B) losing 14000 PLN in a week. The amount of loss in column A increased in consecutive rows (Fig. 2a).

The logic of the pain discounting procedure was very similar to the delay discounting of monetary outcomes. Prior to performing the task, the participants were instructed that they should imagine severe pain with a magnitude of 9 out of 10 that would be constant and unavoidable for a given duration. Such pain intensity, used in previous studies^9^, ensured that the imagined pain would be very strong but not extreme, as might be experienced in a real-life situations concerning chronic pain. In this procedure, participants chose between options A and B. The first column contained 20 rows, which presented option A and began with a choice of no pain now to pain lasting for 14 days beginning from now. The second column contained a fixed option (option B) of pain lasting for 14 days, beginning after a specified delay. For example, the first choice in the shortest delay was set to (A) no pain now or (B) pain lasting for 14 days beginning in one week. The duration of pain in column A increased in consecutive rows (Fig. 2b). For more details please see the online supplementary materials file.

#### Pain anxiety symptom scale

The Pain Anxiety Symptom Scale (PASS) is a self-report measure consisting of 40 items which are rated on a 6-point scale (from 0 = never to 5 = always) and assess symptoms of fear and anxiety associated with pain^21^. In the present research, we focused on the general PASS score, which reflects general pain anxiety and fear of pain.

### Data analysis

The main dependent variables used to measure the steepness of monetary and pain discounting were computed as the areas under the indifference points (Area Under the Curve, AuC)^40^. We assumed that the approximation of an indifference point (i.e., a point that indicates the subjective value of a delayed monetary loss or pain) was equal to the last monetary value or duration of pain chosen from Column A, which contained the present estimates of delayed monetary losses or pain. Overall, in each condition (monetary or pain discounting) we assessed five indifference points that corresponded to five delays in each condition. When the indifference points were plotted as a function of delay (both axes normalized to have values between 0 and 1) and connected with line segments, they formed five trapezoids (see supplementary materials file for graphical presentation of indifference points in CP and Control groups). The sums of their areas constitute the AuC measure, which in effect ranges from 1 (no discounting, exclusive preference for immediate punishment or loss) to 0 (steepest discounting, exclusive preference for delayed punishment or loss). Steeper discounting (lower AuC) means that a given delay discounts (subtracts) more subjective value of an outcome in comparison to a situation where discounting is described as shallower.

Data processing and analysis were performed using the R environment for statistical computing. We used Pearson’s correlation coefficient to examine the association between monetary and pain discounting, and *t*-tests were used to determine whether experiencing CP was related to impulsivity, as measured by AuC. However, because such analyses may give a false picture of results because they omit some of the measured variables in the analysis, a path model was fitted to the data to more accurately describe the relationships under study. Because we had no prior hypotheses regarding strong causality (no relationship between certain variables), a saturated model including CP, age, PASS score and discounting measures was fitted to the data. The analysis was conducted using the Lavaan R package (version 0.6–11) with a diagonally weighted least-squares estimator and bootstrapped standard errors (5000 samples). The PASS subscales were calculated by summing the relevant items, whose scores were used to compute the general score based on factor analysis using regression scores, as recommended by Skrondal and Laake^41^. As no a priori power analysis was conducted, we decided to conduct a post-hoc simulation to estimate power. This analysis was run using a simplified path analysis model which omitted age in the equation. In the simulation we assumed our population parameters to be equal to the values of our empirical standardized estimates, reduced by 10%. Given the population model, the data were simulated 1,000 times for a sample size of N = 255. To assess power for each coefficient, we calculated the percentage of times the estimate was significantly different from 0. Given our assumptions, this simulation indicated that we would have achieved power above 80% threshold for only two paths (pain to PASS: 100%; pain to AuC pain: 99.1%). For all other paths, we would have achieved power lower than 80% (pain to AuC monetary: 35%, PASS to AuC monetary: 76.4%, PASS to AuC pain: 53.5%). Of course, such an analysis is based on assumptions that in no way can be verified by observed data. We decided to report them, however, to point out that low power is one of the possible limitations of the study.

### Supplementary Information

Supplementary Information 1.

## Data Availability

The program codes used in analysis and the data sets generated and/or analyzed during the current study are available online as the supplementary materials file.
