# Supplementary material for: The role of chronic pain and pain anxiety in delay discounting of pain and monetary losses
Source: Sci Rep. 2023 Nov 6;13:19169. doi: 10.1038/s41598-023-46378-4 (PMC10628274; doi:10.1038/s41598-023-46378-4)
Supplement: Supplementary file 1 — Supplementary Information 1. [file 41598_2023_46378_MOESM1_ESM.zip › Supplementary materials.docx]

**Supplementary materials file**

**The Role of Chronic Pain and Pain Anxiety in Delay Discounting of Pain and Monetary Losses**

Wojciech Białaszek^1^*, Szymon Mizak^1^, Paweł Ostaszewski^1^, Przemysław Bąbel^2^

^1^SWPS University Institute of Psychology, DecisionLab: Center for Behavioral Research in Decision Making, Warsaw, Poland

^2^Jagiellonian University, Institute of Psychology, Pain Research Group, Kraków, Poland

*Corresponding author: SWPS University, Institute of Psychology, DecisionLab: Center for Behavioral Research in Decision Making, 03-815 Warsaw, Chodakowska 19/31, Poland, wbialaszek@swps.edu.pl, www.swps.pl

This file contains supplementary materials for the main manuscript.

**Appendix 1a.** Choice questionnaire, financial losses (Polish, original version)

**Appendix 1b.** Choice questionnaire, pain (English translation)

**Appendix 2a.** Choice questionnaire, financial losses (Polish, original version)

**Appendix 2b.** Choice questionnaire, pain (English translation)

**Appendix 3. Figure s1.** Indifference points across two domains (financial losses, pain) in a CP and a control groups.

# Appendix 1a

# KWESTIONARIUSZ DOKONYWANIA WYBORÓW – STRATY FINANSOWE

Kwestionariusz ten służy do badania oceniania wartości strat finansowych. Poprosimy Pana/Panią o dokonanie serii wyborów między różnymi kwotami pieniędzy. Prosimy o wyobrażenie sobie możliwości wyboru między dwoma nieuchronnymi stratami finansowymi. Wybierając jedną z nich, unika się drugiej z danej pary.

Wybory są hipotetyczne, tzn. nie straci Pan/Pani naprawdę kwot, między którymi będzie Pan/Pani wybierać. Tym niemniej prosimy o wskazywanie swoich preferencji tak, jak gdyby przedstawiane sumy miałby/miałaby Pan/Pani naprawdę utracić. Z dwojga złego, którą opcję by Pan/Pani wybrał(a)?

Na kolejnych stronach zobaczy Pan/Pani „Opcję A” po lewej stronie oraz „Opcję B” po prawej stronie.

# Opcja A oznacza, że wybiera Pan/Pani stratę pieniędzy natychmiast. Przy kolejnych wyborach zmieniać się będą kwoty pieniędzy, ale niezmienny pozostanie moment ich straty, tzn. straci Pan/Pani pieniądze od razu.

**Opcja B oznacza, że wybiera Pan/Pani stratę 14 000 zł, ale strata tej kwoty nie będzie natychmiastowa, lecz odsunięta w czasie. Na kolejnych stronach zmieniać się będzie czas, po którym nastąpi strata, ale niezmienna pozostanie kwota straty, tj. 14 000 zł.**

**Uwaga: Niezależnie od wybranej opcji, straty nie będzie można uniknąć.** Innymi słowy, niezależnie od tego, czy wybierze Pan/Pani stratę 5 000 zł od razu czy 14 000 zł za miesiąc, straty tej nie będzie można uniknąć.

Prosimy Pana/Panią o dokonywanie wyborów poprzez **otoczenie kółkiem** jednej z dwóch opcji – “A” lub “B”, tak jak wyjaśniamy to poniżej w zadaniu przykładowym. Wybierając jedną z opcji, unika się drugiej z danej pary. **Prosimy o zwrócenie uwagi, że na kolejnych stronach zmieniać się będzie czas, po jakim doszłoby do utraty pieniędzy w ramach opcji „B”.**

Interesują nas Pana/Pani preferencje. Nie ma tu ani dobrych, ani złych wyborów. Nie mamy żadnych oczekiwań wobec Pana/Pani, z wyjątkiem tego, by wybierał(a) Pan/Pani zgodnie z własnymi przekonaniami. Im bardziej szczere będą Pana/Pani decyzje, tym większą wartość naukową będą miały rezultaty naszego badania! Badanie jest anonimowe, a jego wyniki analizowane będą na poziomie grupowym, a nie indywidualnym.

Prosimy o dokonywanie wyborów po kolei.

Prosimy dokonać wszystkich wyborów na każdej ze stron i nie powracać do raz podjętych decyzji.

# Przykład:

A: 1000 zł **od razu** B: 14 000 zł **za tydzień**


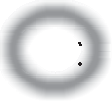
Jeżeli wolałby/wolałaby Pan/Pani stracić 1 000 zł **od razu**, to należałoby zaznaczyć „**A**”.

A: 1000 zł **od razu** B: 14 000 zł **za tydzień**


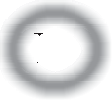
Jeżeli wolałby/wolałaby Pan/Pani stracić 14 000 zł **za tydzień**, to należałoby zaznaczyć „**B**”.

A: 1000 zł **od razu** B: 14 000 zł **za tydzień**

Strata od razu lub **za tydzień***

| **Opcja A** | | **Opcja B** | |
| --- | --- | --- | --- |
| A: | 0 zł od razu | B: | 14 000 zł za tydzień* |
| A: | 50 zł od razu | B: | 14 000 zł za tydzień |
| A: | 100 zł od razu | B: | 14 000 zł za tydzień |
| A: | 150 zł od razu | B: | 14 000 zł za tydzień |
| A: | 300 zł od razu | B: | 14 000 zł za tydzień |
| A: | 500 zł od razu | B: | 14 000 zł za tydzień |
| A: | 1000 zł od razu | B: | 14 000 zł za tydzień |
| A: | 2000 zł od razu | B: | 14 000 zł za tydzień |
| A: | 3000 zł od razu | B: | 14 000 zł za tydzień |
| A: | 4000 zł od razu | B: | 14 000 zł za tydzień |
| A: | 5000 zł od razu | B: | 14 000 zł za tydzień |
| A: | 6000 zł od razu | B: | 14 000 zł za tydzień |
| A: | 7000 zł od razu | B: | 14 000 zł za tydzień |
| A: | 8000 zł od razu | B: | 14 000 zł za tydzień |
| A: | 9000 zł od razu | B: | 14 000 zł za tydzień |
| A: | 10 000 zł od razu | B: | 14 000 zł za tydzień |
| A: | 11 000 zł od razu | B: | 14 000 zł za tydzień |
| A: | 12 000 zł od razu | B: | 14 000 zł za tydzień |
| A: | 13 000 zł od razu | B: | 14 000 zł za tydzień |
| A: | 14 000 zł od razu | B: | 14 000 zł za tydzień |

*Note: this questionnaire contained four more pages, where „za tydzień” [in a week] was substituted respectively by: za miesiąc, za rok, za 5 lat, za 15 lat.

# Appendix 1b

# CHOICE QUESTIONNAIRE – FINANCIAL LOSSES

This questionnaire is used to assess the value of financial losses. We will ask you to make a series of choices between different amounts of money. Please imagine having the option to choose between two inevitable financial losses. By choosing one of them, you avoid the other in the pair.

The choices are hypothetical, meaning, you will not actually lose the amounts between which you will be choosing. Nonetheless, we ask you to indicate your preferences as if you really had to incur the losses presented. Between the two unfavorable options, which one would you choose?

On the following pages, you will see “Option A” on the left side and “Option B” on the right side.

**Option A means that you choose a financial loss immediately. In subsequent choices, the amounts of money will change, but the delay of the loss will remain the same; that is, you will lose the money immediately.**

**Option B means that you choose a loss of 14,000 PLN, but this loss will not be immediate but will be delayed. On the following pages, the delay of the loss will change, but the amount of loss, i.e., 14,000 PLN, will remain the same.**

**Note: Regardless of the chosen option, the loss cannot be avoided.** In other words, whether you choose an immediate loss of 5,000 PLN or a loss of 14,000 PLN in a month, this loss cannot be avoided.

We ask you to make your choices **by circling** one of the two options – “A” or “B”, as explained in the example task below. By choosing one of the options, you avoid the other in the pair. **Please note that on the following pages, the delay of the monetary loss within option “B” will change.**

We are interested in your preferences. There are no right or wrong choices here. We have no expectations of you, except that you choose according to your own beliefs. The more sincere your decisions are, the greater the scientific value our research results will have! The study is anonymous, and its results will be analyzed at the group level, not the individual level.

Please make your choices one by one.

Please make all choices on each page and do not go back to change decisions once made.

**Example:**

A: 1000 PLN **now** B: 14 000 PLN **in a week**

If you prefer to lose 1,000 PLN immediately, select **'A'**.
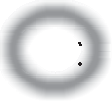


A: 1000 PLN **now** B: 14 000 zł **in a week**


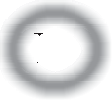
If you prefer to lose 14,000 PLN in a week, select **'B'**.

A: 1000 PLN **now** B: 14 000 PLN **in a week**

Loss now or **in** **a week***

| **Opcja A** | | **Opcja B** | |
| --- | --- | --- | --- |
| A: | 0 PLN now | B: | 14 000 PLN in a week |
| A: | 50 PLN now | B: | 14 000 PLN in a week |
| A: | 100 PLN now | B: | 14 000 PLN in a week |
| A: | 150 PLN now | B: | 14 000 PLN in a week |
| A: | 300 PLN now | B: | 14 000 PLN in a week |
| A: | 500 PLN now | B: | 14 000 PLN in a week |
| A: | 1000 PLN now | B: | 14 000 PLN in a week |
| A: | 2000 PLN now | B: | 14 000 PLN in a week |
| A: | 3000 PLN now | B: | 14 000 PLN in a week |
| A: | 4000 PLN now | B: | 14 000 PLN in a week |
| A: | 5000 PLN now | B: | 14 000 PLN in a week |
| A: | 6000 PLN now | B: | 14 000 PLN in a week |
| A: | 7000 PLN now | B: | 14 000 PLN in a week |
| A: | 8000 PLN now | B: | 14 000 PLN in a week |
| A: | 9000 PLN now | B: | 14 000 PLN in a week |
| A: | 10 000 PLN now | B: | 14 000 PLN in a week |
| A: | 11 000 PLN now | B: | 14 000 PLN in a week |
| A: | 12 000 PLN now | B: | 14 000 PLN in a week |
| A: | 13 000 PLN now | B: | 14 000 PLN in a week |
| A: | 14 000 PLN now | B: | 14 000 PLN in a week |

*Note: this questionnaire contained four more pages, where „in a week” was substituted respectively by: in a month, in a year, in 5 years, in 15 years.

# Apendix 2a

# KWESTIONARIUSZ DOKONYWANIA WYBORÓW - BÓL

Kwestionariusz ten służy do badania dokonywania specyficznych wyborów: czasu trwania bólu i momentu, w którym ból będzie doświadczany. Prosimy sobie wyobrazić, że ma Pan/Pani możliwość dokonania wyboru czasu trwania i momentu odczuwania bólu. Każdy człowiek doświadcza w swoim życiu tego doznania. Prosimy wyobrazić sobie ten rodzaj bólu, który dobrze Pan/Pani zna. Może to być np. ból głowy, ból zęba, ból pleców czy inny.

Wybory są hipotetyczne, tzn. nie będzie Pan/Pani w rzeczywistości odczuwać bólu trwającego tyle czasu i rozpoczynającego się w momencie, który Pan/Pani wybrał/a. Tym niemniej prosimy Pana/Panią o wybieranie tak, jak gdyby przedstawiane możliwości były realne. Z dwojga złego, którą opcję by Pan/Pani wybrał(a)?

Na kolejnych stronach zobaczy Pan/Pani „Opcję A” po lewej stronie oraz „Opcję B” po prawej stronie.

# Opcja A oznacza, że wybiera Pan/Pani odczuwanie bólu od teraz, natychmiast. Przy kolejnych wyborach będzie się zmieniać czas trwania bólu, ale niezmienny będzie moment jego rozpoczęcia, tzn. ból będzie odczuwany od teraz.

**Opcja B oznacza, że wybiera Pan/Pani odczuwanie bólu rozpoczynające się po upływie określonego czasu. Na kolejnych stronach odroczenie bólu będzie się zmienić, ale niezmienny pozostanie czas jego trwania.**

**Uwaga:**

1. **Niezależnie od wybranej opcji ból będzie równie silny. Różnić się będzie tylko czas jego trwania.** Siłę bólu w każdym przypadku oszacować można jako 9 na skali od 0 do 10. Innymi słowy, niezależnie od tego, czy wybierze Pan/Pani np. ból trwający przez godzinę, a rozpoczynający się od teraz czy trwający przez 14 dni, ale rozpoczynający się za tydzień, ból ten będzie miał taką samą, stałą siłę.
2. **W każdym przypadku ból będzie mieć charakter ciągły, tzn. ból odczuwany będzie bez przerwy**, niezależnie od tego, czy będzie trwać przez godzinę czy przez 14 dni.
3. **Niezależnie od wybranej opcji, bólu nie będzie można uniknąć.** Innymi słowy, niezależnie od tego, czy wybierze Pan/Pani np. ból trwający przez godzinę, a rozpoczynający się od teraz czy trwający przez 14 dni, ale rozpoczynający się za tydzień, bólu nie będzie można uniknąć.

Prosimy Pana/Panią o dokonywanie wyborów poprzez **otoczenie kółkiem** jednej z dwóch opcji – “A” lub “B”, tak jak wyjaśniamy to poniżej w zadaniu przykładowym. Wybierając jedną z opcji, unika się drugiej z danej pary. **Prosimy o zwrócenie uwagi, że na kolejnych stronach zmieniać się będzie czas, po jakim odczuwałby/odczuwałaby Pan/Pani ból w ramach opcji "B".**

Interesują nas Pana/Pani preferencje. Nie ma tu ani dobrych, ani złych wyborów. Nie mamy żadnych oczekiwań wobec Pana/Pani, z wyjątkiem tego, by wybierał(a) Pan/Pani zgodnie z własnymi przekonaniami. Im bardziej szczere będą Pana/Pani decyzje, tym większą wartość naukową będą miały rezultaty naszego badania! Badanie jest anonimowe, a jego wyniki analizowane będą na poziomie grupowym, a nie indywidualnym.

Prosimy o dokonywanie wyborów po kolei.

Prosimy dokonać wszystkich wyborów na każdej ze stron i nie powracać do raz podjętych decyzji.

# Przykład:

A: ból trwający 8 godzin **od teraz** B: ból trwający 14 dni **za tydzień**


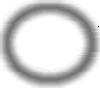
Jeżeli wolałby/wolałaby Pan/Pani doświadczyć silnego bólu trwającego nieprzerwanie przez 8 godzin, zaczynającego się **od razu**, to należałoby zaznaczyć „**A**”.

A: ból trwający 8 godzin **od teraz** B: ból trwający 14 dni **za tydzień**


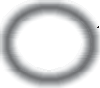
Jeżeli wolałby/wolałaby Pan/Pani doświadczyć silnego bólu trwającego nieprzerwanie przez 14 dni, ale rozpoczynającego się **za tydzień**, to należałoby zaznaczyć „**B**”.

A: ból trwający 8 godzin **od teraz** B: ból trwający 14 dni **za tydzień**

Silny ból od teraz lub równie silny ból **rozpoczynający się za tydzień***

| **Opcja A** | | **Opcja B** | |
| --- | --- | --- | --- |
| A: | brak bólu od teraz | B: | ból trwający 14 dni za tydzień |
| A: | ból trwający 1 godzinę od teraz | B: | ból trwający 14 dni za tydzień |
| A: | ból trwający 2 godziny od teraz | B: | ból trwający 14 dni za tydzień |
| A: | ból trwający 4 godziny od teraz | B: | ból trwający 14 dni za tydzień |
| A: | ból trwający 8 godzin od teraz | B: | ból trwający 14 dni za tydzień |
| A: | ból trwający 12 godzin od teraz | B: | ból trwający 14 dni za tydzień |
| A: | ból trwający 24 godzin od teraz | B: | ból trwający 14 dni za tydzień |
| A: | ból trwający 2 dni od teraz | B: | ból trwający 14 dni za tydzień |
| A: | ból trwający 3 dni od teraz | B: | ból trwający 14 dni za tydzień |
| A: | ból trwający 4 dni od teraz | B: | ból trwający 14 dni za tydzień |
| A: | ból trwający 5 dni od teraz | B: | ból trwający 14 dni za tydzień |
| A: | ból trwający 6 dni od teraz | B: | ból trwający 14 dni za tydzień |
| A: | ból trwający 7 dni od teraz | B: | ból trwający 14 dni za tydzień |
| A: | ból trwający 8 dni od teraz | B: | ból trwający 14 dni za tydzień |
| A: | ból trwający 9 dni od teraz | B: | ból trwający 14 dni za tydzień |
| A: | ból trwający 10 dni od teraz | B: | ból trwający 14 dni za tydzień |
| A: | ból trwający 11 dni od teraz | B: | ból trwający 14 dni za tydzień |
| A: | ból trwający 12 dni od teraz | B: | ból trwający 14 dni za tydzień |
| A: | ból trwający 13 dni od teraz | B: | ból trwający 14 dni za tydzień |
| A: | ból trwający 14 dni od teraz | B: | ból trwający 14 dni za tydzień |

*Note: this questionnaire contained four more pages, where „za tydzień” [in a week] was substituted respectively by: za miesiąc, za rok, za 5 lat, za 15 lat.

# Appendix 2b

# CHOICE QUESTIONNAIRE - PAIN

This questionnaire aims to study specific choices: the duration of pain and the moment at which the pain will be experienced. Please imagine that you have the opportunity to choose the duration and onset of pain. Everyone experiences this sensation in their life. Please visualize the type of pain that you are well acquainted with. It could be, for example, a headache, toothache, back pain, or another type.

Wybory są hipotetyczne, tzn. nie będzie Pan/Pani w rzeczywistości odczuwać bólu trwającego tyle czasu i rozpoczynającego się w momencie, który Pan/Pani wybrał/a. Tym niemniej prosimy Pana/Panią o wybieranie tak, jak gdyby przedstawiane możliwości były realne. Z dwojga złego, którą opcję by Pan/Pani wybrał(a)?

The choices are hypothetical, meaning you will not actually experience the pain lasting for the chosen duration or starting at the chosen moment. Nevertheless, please make your choices as if the presented options were real. Which option is the lesser of two evils?

On the following pages, you will see "Option A" on the left and "Option B" on the right.

# Option A indicates choosing to experience pain from now, immediately. In subsequent choices, the duration of the pain will vary, but the onset will remain constant; that is, the pain will be experienced from now on.

**Option B indicated choosing to experience pain that will start after a specified period. On the following pages, the delay in the onset of pain will change, but the duration will remain the same.**

**Note:**

1. **Regardless of the selected option, the pain will be equally intense. Only its duration will differ.** The intensity of the pain can be estimated as 9 on a scale from 0 to 10. In other words, whether you choose, for example, pain lasting for an hour starting now or pain lasting for 14 days but starting a week later, the pain will have the same, constant intensity.
2. **In all cases, the pain will be continuous; that is, the pain will be felt constantly,** regardless of whether it lasts for an hour or for 14 days.
3. **Regardless of the selected option, the pain cannot be avoided.** In other words, whether you choose, for example, pain lasting for an hour starting now or pain lasting for 14 days but starting a week later, the pain cannot be avoided.

Please make your choices **by circling** one of the two options—"A" or "B," as explained below in the example task. By choosing one of the options, you are avoiding the other in the given pair. **Please note that on the following pages, the time after which you would feel the pain under option "B" will change.**

We are interested in your preferences. There are no right or wrong choices here. We have no expectations from you, other than for you to make selections according to your own convictions. The more sincere your decisions, the greater the scientific value our study will hold! The study is anonymous, and its results will be analyzed at the group level, not individually.

We are interested in your preferences. There are no right or wrong choices here. We have no expectations of you, except that you choose according to your own beliefs. The more sincere your decisions are, the greater the scientific value our research results will have! The study is anonymous, and its results will be analyzed at the group level, not the individual level.

Please make your choices one by one.

Please make all choices on each page and do not go back to change decisions once made.

**Example:**

A: pain lasting for 8 hours **from now** B: pain lasting 14 dni **in a week**

If you would prefer to experience intense pain lasting continuously for 8 hours, starting **immediately**, you should select "A."

A: pain lasting for 8 hours **from now** B: pain lasting 14 dni **in a week**

If you would prefer to experience intense pain lasting continuously for 14 days, but starting a week later, you should select "B."
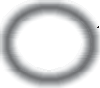


A: pain lasting for 8 hours **from now** B: pain lasting 14 dni **in a week**

Severe pain now or equally severe pain beginning **in a week***

| **Opcja A** | | **Opcja B** | | |
| --- | --- | --- | --- | --- |
| A: | no pain from now | B: | pain lasting 14 days in a week* |  |
| A: | pain lasting 1 hour from now | B: | pain lasting 14 days in a week |  |
| A: | pain lasting 2 hours from now | B: | pain lasting 14 days in a week |  |
| A: | pain lasting 4 hours from now | B: | pain lasting 14 days in a week |  |
| A: | pain lasting 8 hours from now | B: | pain lasting 14 days in a week |  |
| A: | pain lasting 12 hours from now | B: | pain lasting 14 days in a week |  |
| A: | pain lasting 24 hours from now | B: | pain lasting 14 days in a week |  |
| A: | pain lasting 2 days from now | B: | pain lasting 14 days in a week |  |
| A: | pain lasting 3 days from now | B: | pain lasting 14 days in a week |  |
| A: | pain lasting 4 days from now | B: | pain lasting 14 days in a week |  |
| A: | pain lasting 5 days from now | B: | pain lasting 14 days in a week |  |
| A: | pain lasting 6 days from now | B: | pain lasting 14 days in a week |  |
| A: | pain lasting 7 days from now | B: | pain lasting 14 days in a week |  |
| A: | pain lasting 8 days from now | B: | pain lasting 14 days in a week |  |
| A: | pain lasting 9 days from now | B: | pain lasting 14 days in a week |  |
| A: | pain lasting 10 days from now | B: | pain lasting 14 days in a week |  |
| A: | pain lasting 11 days from now | B: | pain lasting 14 days in a week |  |
| A: | pain lasting 12 days from now | B: | pain lasting 14 days in a week |  |
| A: | pain lasting 13 days from now | B: | pain lasting 14 days in a week |  |
| A: | pain lasting 14 days from now | B: | pain lasting 14 days in a week |  |

*Note: this questionnaire contained four more pages, where „in a week” was substituted respectively by: in a month, in a year, in 5 years, in 15 years.

# Appendix 3


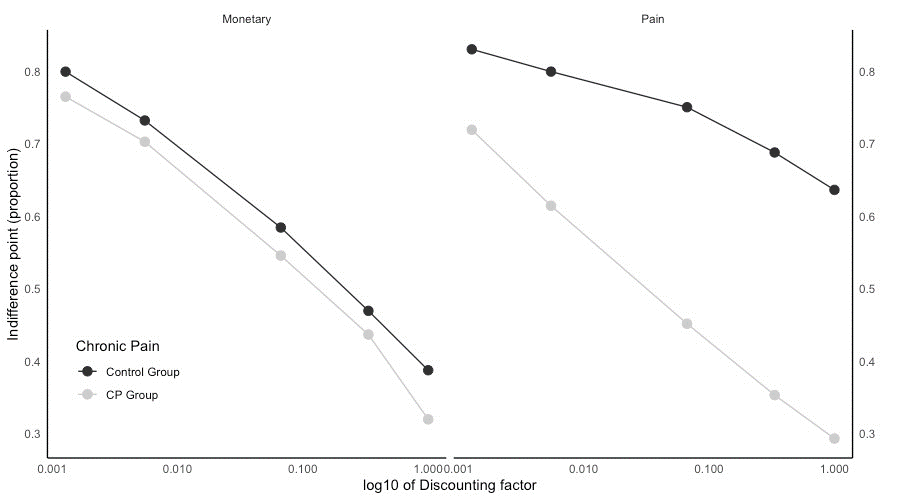


**Figure s1.** The mean subjective value of the monetary loss (left) and the duration of pain (right), in the CP and the Control Group, expressed as indifference points corresponding to five delays: 1 week, 1 month, 1 year, 5 years, and 15 years. Both axes are normalized to have values between 0 and 1. Note: the horizontal axis, representing delay in days, was log_10_ transformed to facilitate the readability of the figure.
